# Delayed Terminal Ileal Obstruction From a Retained Percutaneous Endoscopic Gastrostomy (PEG) Bolster Following Cut-and-Push Replacement in a Frail Elderly Patient: A Case Report

**DOI:** 10.7759/cureus.109604

**Published:** 2026-05-25

**Authors:** Zafar I Gondal, Ameirah B Alali, Aleena Chaudhary, Muhammad Bilal, Dima Al-Qaimari, Sanar Al-Qaimari, Rana T Hamada

**Affiliations:** 1 Department of General Surgery, Sheikh Khalifa Hospital, Fujairah, ARE; 2 Department of Gastroenterology, Sheikh Khalifa Hospital, Fujairah, ARE; 3 College of Medicine, University of Sharjah, Sharjah, ARE

**Keywords:** adhesions, elderly patient, enteral feeding, laparotomy, minimally invasive surgery, peg tube, retained bolster, small bowel obstruction, terminal ileum

## Abstract

Percutaneous endoscopic gastrostomy (PEG) is commonly used for long-term enteral nutrition in patients unable to feed orally. The cut-and-push technique, where the internal bolster is left to pass spontaneously, is generally safe, but complications are uncommon. We report an 88-year-old bed-bound female with multiple comorbidities, including type 2 diabetes mellitus, chronic kidney disease, dementia, and a prior lower midline laparotomy, who underwent PEG replacement using the cut-and-push method. Three weeks later, she developed a small bowel obstruction. Imaging revealed the retained PEG bolster lodged in the terminal ileum, causing proximal dilatation and distal collapse. Following a 48-hour trial of conservative management without clinical improvement, minimally invasive intervention was attempted. Diagnostic laparoscopy with adhesiolysis was initially performed, followed by an attempted colonoscopic retrieval; however, colonoscopy was unsuccessful because of inadequate bowel preparation. Definitive management required open surgical retrieval through enterotomy via the previous laparotomy scar, which was completed successfully. Postoperatively, the patient required prolonged ventilatory support via tracheostomy and was transferred to a long-term care facility. This case illustrates a rare delayed obstruction from a retained PEG bolster, highlighting the importance of careful patient selection, close follow-up to confirm spontaneous passage, and timely intervention when excretion fails.

## Introduction

Percutaneous endoscopic gastrostomy (PEG) is a widely accepted and effective method for providing long-term enteral nutrition in patients who are unable to maintain adequate oral intake, particularly those with neurological disorders, malignancies, or chronic systemic illness. Owing to its minimally invasive nature, PEG has become the preferred modality over surgical gastrostomy, offering favorable safety, efficacy, and cost profiles [1,2].

Despite its overall safety, PEG tube-related complications can occur, especially during tube replacement. Common indications for replacement include tube dysfunction, blockage, or accidental dislodgement. In cases involving PEG tubes with an internal retention bolster, replacement can be performed either through endoscopic retrieval or via the *cut-and-push *technique, whereby the external portion of the tube is cut, and the internal bumper is left to pass spontaneously through the gastrointestinal tract [2,3].

The cut-and-push method represents a simple, minimally invasive, and cost-effective alternative to endoscopic removal, as it avoids the need for sedation and specialized procedural resources. In this technique, the internal PEG bumper is detached by cutting the external tube at skin level and subsequently passes through the gastrointestinal tract by normal bowel peristalsis and stool transit until it is naturally excreted. Previous studies have reported high rates of spontaneous passage of the internal bumper, ranging from approximately 84% to 97%, with relatively low complication rates [2,3]. However, this approach is not without risk. Retained internal bumpers may lead to serious complications, including bowel obstruction, perforation, or impaction.

Although generally considered safe, rare delayed complications following the cut-and-push technique have been described, particularly in patients with impaired gastrointestinal transit. The present case is particularly noteworthy as it describes a delayed terminal ileal small bowel obstruction caused by a retained PEG bolster in a frail elderly patient following PEG replacement. This case highlights the potential for significant late complications even in techniques considered safe and minimally invasive, while also emphasizing the importance of appropriate patient selection and follow-up.

Importantly, certain patient-related factors increase the risk of adverse outcomes, including prior abdominal surgery, intra-abdominal adhesions, gastrointestinal strictures, and impaired bowel motility. Such conditions may interfere with normal intestinal transit and reduce the likelihood of spontaneous passage of the retained internal bolster. Notably, the patient in the present case had a history of prior lower midline laparotomy, a factor associated with an increased risk of adhesions and impaired intestinal transit. In such cases, failure of spontaneous passage is more likely, and alternative strategies such as endoscopic removal may be more appropriate [3].

Given the absence of universally standardized guidelines, careful patient selection and risk stratification are essential when considering the cut-and-push technique. A thorough understanding of its benefits, mechanism, and potential limitations is therefore critical to ensuring patient safety and optimizing clinical outcomes.

## Case presentation

An 88-year-old bed-bound female on long-term home oxygen therapy (2 L/minute via nasal cannula) was admitted to the intensive care unit (ICU) following an episode of breakthrough seizures requiring acute stabilization and close neurological monitoring. Her past medical history was significant for diabetes mellitus, hypertension, dyslipidemia, chronic kidney disease, seizure disorder, recurrent transient ischemic attacks, dementia, asthma, and a previous lower midline laparotomy.

On Day 1 of admission, she underwent replacement of a blocked percutaneous endoscopic gastrostomy (PEG) tube. Given her extreme frailty, multiple comorbidities, and very high anesthetic risk, endoscopic retrieval of the internal bumper was considered high-risk. After multidisciplinary assessment, the cut-and-push technique was selected as the least invasive and most appropriate option to avoid sedation, endoscopy-related complications, and procedural instability in an ICU setting. The procedure was performed by the gastroenterology team at Sheikh Khalifa Hospital, Fujairah (SKHF). Her post-procedural course was initially uneventful, and she was discharged after three days.

Three weeks after PEG replacement, the patient developed progressive abdominal distension, colicky abdominal pain, persistent vomiting, and reduced bowel movements, raising suspicion of intestinal obstruction. There was no passage of stool or flatus during the preceding days. On examination, she had a markedly distended abdomen with generalized tenderness without guarding or rigidity. Bowel sounds were hyperactive and high-pitched. Laboratory investigations showed mild leukocytosis without significant electrolyte imbalance or metabolic derangement.

Contrast-enhanced computed tomography (CT) of the abdomen demonstrated a partial small bowel obstruction caused by a retained PEG tube cuff lodged in a loop of the terminal ileum, with proximal jejunal and ileal dilatation and collapse of the distal bowel (Figure 1). The patient had initially undergone conservative management at another facility without improvement and was subsequently referred to SKHF for further management.

**Figure 1:**
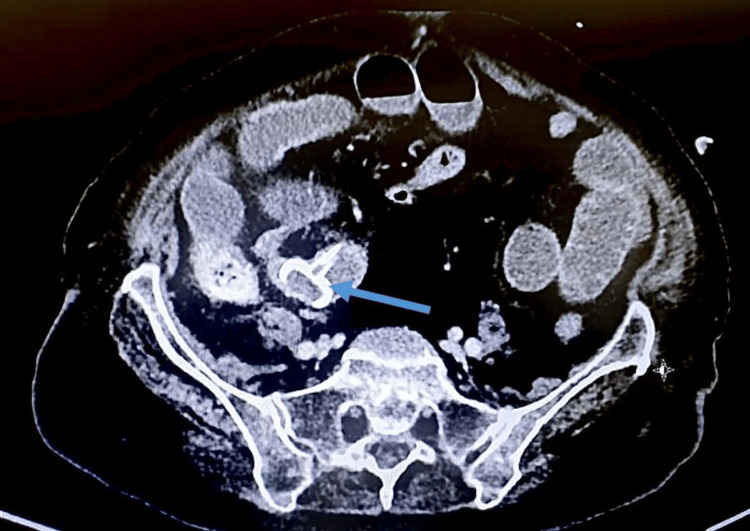
Contrast-enhanced computed tomography (CT) showing a retained percutaneous endoscopic gastrostomy (PEG) bolster causing small bowel obstruction. Contrast-enhanced CT scan of the abdomen demonstrates a retained PEG bolster lodged in the terminal ileum (blue arrow). There is associated proximal small bowel dilatation with distal bowel collapse, consistent with mechanical small bowel obstruction. These findings are consistent with obstruction secondary to impaction of the PEG component in the terminal ileum.

A multidisciplinary team discussion involving gastroenterology, general surgery, and anesthesia services concluded that a stepwise minimally invasive strategy would be most appropriate given her frailty and comorbidities. The plan included diagnostic laparoscopy with attempted *milking* of the retained PEG component toward the ileocecal junction (ICJ), followed by colonoscopic retrieval, aiming to avoid enterotomy. This combined approach was chosen to minimize surgical trauma while improving the likelihood of successful endoscopic extraction. Although bowel preparation is generally preferred for colonoscopic retrieval, it was not feasible due to the acute obstructive presentation, where bowel cleansing was contraindicated and impractical.

On Day 24, diagnostic laparoscopy revealed localized adhesions from her previous lower midline laparotomy, resulting in kinking of a terminal ileal loop near the ICJ with the PEG cuff trapped at the site of obstruction. Adhesiolysis was performed, relieving the kink and restoring bowel alignment. Subsequent colonoscopy was attempted; however, it was unsuccessful due to poor visualization from fecal loading of the caecum and terminal ileum, related to the absence of bowel preparation in the emergency setting.

Given the failure of endoscopic retrieval and ongoing obstruction, and after reassessment by the anesthesia and surgical teams to minimize operative duration and risk, the decision was made to proceed with open surgical removal. An enterotomy was performed through the previous laparotomy scar, and the retained PEG component was successfully extracted (Figures 2-3). The patient was transferred postoperatively to the ICU, where she was extubated on Day 28. However, she required reintubation on Day 29 due to respiratory compromise secondary to poor respiratory reserve and critical illness weakness. Despite repeated attempts at weaning, extubation was unsuccessful, and a tracheostomy was performed on Day 42. She was subsequently transferred to a long-term care facility on ventilatory support.

**Figure 2:**
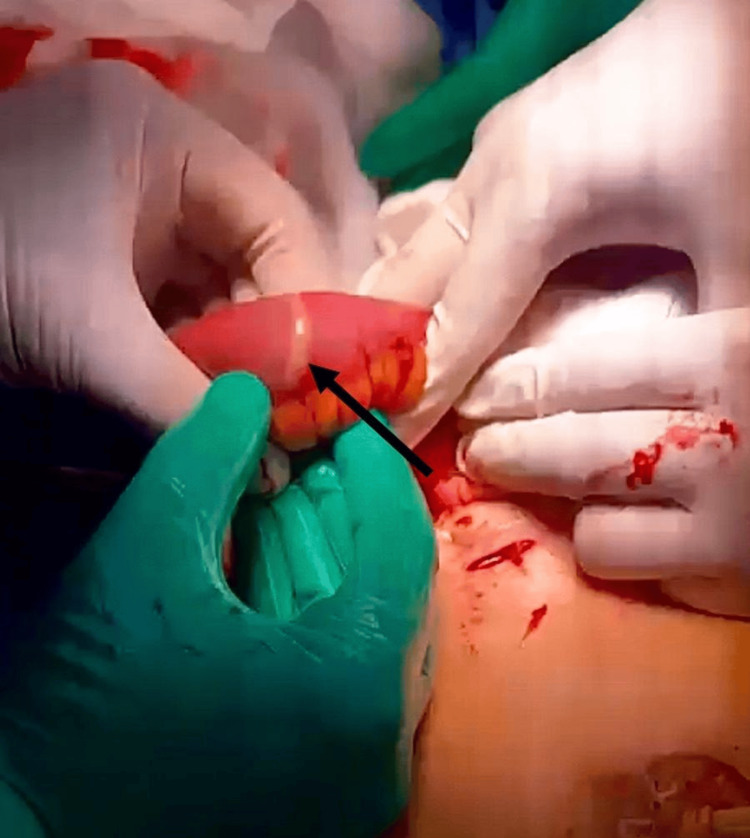
Intraoperative view of a retained percutaneous endoscopic gastrostomy (PEG) bolster in the terminal ileum. Intraoperative view demonstrating a retained PEG bolster lodged within a loop of the terminal ileum (black arrow). The surrounding small bowel loops show adhesions causing kinking of the affected segment. Adhesiolysis was performed to release the kink and restore bowel alignment prior to attempted colonoscopic retrieval of the retained PEG component.

**Figure 3:**
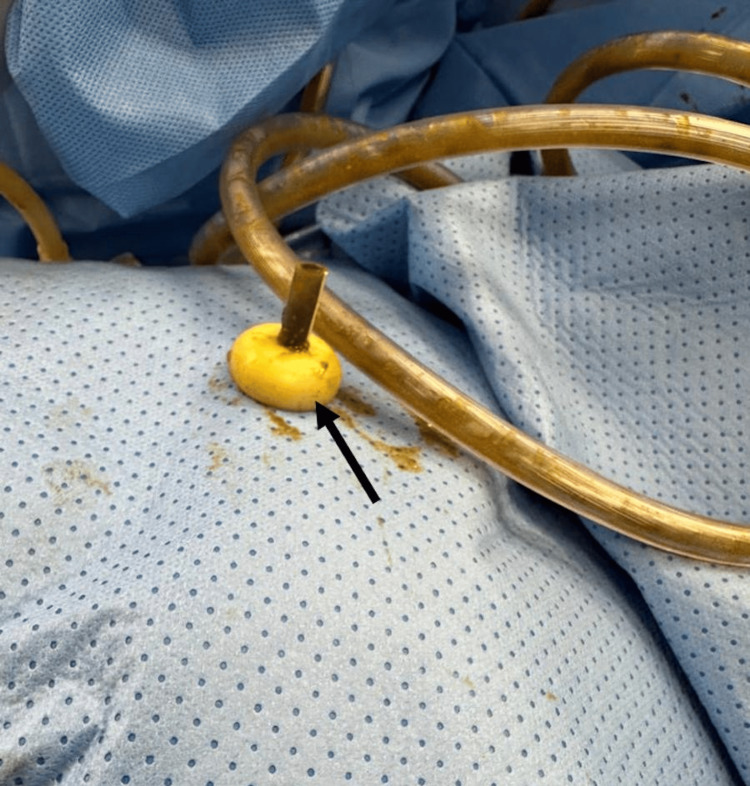
Retrieved percutaneous endoscopic gastrostomy (PEG) bolster following surgical enterotomy. Postoperative image demonstrates the retrieved PEG bolster following surgical removal via enterotomy (black arrow). The extracted device is intact, confirming successful removal of the foreign body responsible for the small bowel obstruction.

## Discussion

PEG is a well-established method for providing long-term enteral nutrition in patients unable to feed orally due to impaired swallowing. PEG tubes are generally classified into two types: balloon-type and internal bolster-type. Balloon-type tubes typically require replacement every three to six months, or according to the manufacturer’s recommendations, whereas routine replacement of internal bolster-type PEG tubes is not performed unless complications such as infection, breakage, dislodgement, occlusion, or leakage occur [4].

The method chosen for PEG tube removal or replacement depends on the type of tube. Percutaneous traction involves releasing the internal fixation plate and extracting the tube externally through the abdominal wall [5]. The cut-and-push technique entails cutting the external portion of the catheter and allowing the internal bolster to pass spontaneously through the gastrointestinal tract [6]. Alternatively, endoscopic retrieval enables removal of the internal component under direct endoscopic visualization [7,8].

In our patient, the cut-and-push technique was used to replace a blocked PEG tube. Although prior lower abdominal surgery is generally considered a relative contraindication due to the risk of adhesions and impaired bowel transit, the decision was made after multidisciplinary discussion because of the patient’s extreme frailty, advanced age, multiple comorbidities, and high anesthetic risk, where avoidance of sedation and endoscopic intervention was prioritized. Endoscopic retrieval was considered but deferred in view of these risks. The expectation was that the internal bolster would be naturally expelled through bowel movements, consistent with published safety data [9,10].

This expectation, while generally appropriate, warrants critical reconsideration in this case. The patient’s prior lower midline laparotomy, together with frailty and likely reduced gut motility, may have significantly reduced the probability of spontaneous passage and increased the risk of retention and impaction. This highlights the importance of individualized risk assessment, particularly when guideline-based contraindications intersect with real-world clinical constraints.

The cut-and-push technique, first introduced in 1991 [11], has been promoted as a cost-effective and resource-saving alternative to endoscopic removal. Reported outcomes demonstrate spontaneous passage in 84%-97% of cases, with complications such as abdominal pain or gastrointestinal obstruction occurring in approximately 1.6%-2.7% [4].

Despite these favorable outcomes, the technique has important limitations. It is generally contraindicated in patients with gastrointestinal strictures, prior abdominopelvic surgery, or impaired gastrointestinal motility, as these factors increase the risk of failure of spontaneous excretion [9]. In the present case, prior abdominal surgery represented a significant risk factor for adhesion formation and impaired bowel transit, which likely contributed to retention of the PEG component and subsequent obstruction. The decision to proceed nonetheless reflects the clinical balance between procedural risk and anesthetic risk in a critically frail patient.

Complications of this nature are rarely reported in the literature. One published case described persistent obstruction requiring endoscopic removal of the retained PEG tube [9], while another reported surgical retrieval of a PEG tube embedded in the abdominal wall [12]. Notably, both cases required intervention within two weeks of the cut-and-push procedure. Current recommendations suggest that if the internal bolster has not been excreted within two weeks, further evaluation and intervention should be considered.

In the present case, although the patient was discharged with standard advice regarding spontaneous passage of the PEG component, no formal follow-up imaging or structured review was arranged to confirm excretion. This represents a limitation in post-procedural monitoring and an important learning point, as earlier detection might have altered the clinical course.

The management of this patient required a staged multidisciplinary approach. Diagnostic laparoscopy was performed to assess adhesions and facilitate the mobilization of the retained PEG component toward the ileocecal junction. This was followed by an attempted colonoscopic retrieval; however, this was unsuccessful due to poor visualization secondary to fecal loading in the absence of bowel preparation, which was not feasible in the acute obstructive setting. Subsequently, open surgical retrieval via enterotomy was required due to failed minimally invasive approaches and the patient’s ongoing obstruction.

To improve clinical practice, this case highlights the potential value of early imaging follow-up or confirmation of passage of the internal PEG component in high-risk patients. It also supports consideration of alternative strategies, such as primary endoscopic retrieval, in patients with significant risk factors, including prior abdominal surgery, even when procedural risk appears higher.

## Conclusions

The cut-and-push technique for PEG tube replacement is generally safe and minimally invasive, with a high rate of spontaneous passage of the internal bumper. However, patients with prior abdominal surgery, intra-abdominal adhesions, or impaired gastrointestinal motility are at increased risk of retained PEG components and subsequent small bowel obstruction.

This case highlights a rare but important delayed complication of the cut-and-push technique, resulting in terminal ileal obstruction requiring escalation from conservative management to laparoscopic adhesiolysis and ultimately open surgical retrieval. It underscores the importance of careful patient selection, early clinical follow-up, and confirmation of passage of the internal PEG component, as well as timely intervention when spontaneous excretion fails.

Importantly, in patients with significant risk factors, particularly prior abdominal surgery, a lower threshold for primary endoscopic retrieval rather than the cut-and-push technique may be warranted to reduce the risk of distal impaction and bowel obstruction.

Awareness of this complication can assist clinicians in risk stratification, procedural planning, and post-procedural monitoring in high-risk patients undergoing PEG tube replacement.
